# Finding common ground to achieve a “good death”: family physicians working with substitute decision-makers of dying patients. A qualitative grounded theory study

**DOI:** 10.1186/1471-2296-14-14

**Published:** 2013-01-22

**Authors:** Amy Tan, Donna Manca

**Affiliations:** 1 Department of Family Medicine, 205 College Plaza, University of Alberta, 8215-112 Street, Edmonton, Alberta, T6G 2C8, Canada; 2 Department of Family Medicine, 901 College Plaza, University of Alberta, 8215-112 Street, Edmonton, Alberta, T6G 2C8, Canada

**Keywords:** Family medicine, Advance care planning, Conflict, Substitute decision-makers, Good death

## Abstract

**Background:**

Substitute decision-makers are integral to the care of dying patients and make many healthcare decisions for patients. Unfortunately, conflict between physicians and surrogate decision-makers is not uncommon in end-of-life care and this could contribute to a “bad death” experience for the patient and family. We aim to describe Canadian family physicians’ experiences of conflict with substitute decision-makers of dying patients to identify factors that may facilitate or hinder the end-of-life decision-making process. This insight will help determine how to best manage these complex situations, ultimately improving the overall care of dying patients.

**Methods:**

Grounded Theory methodology was used with semi-structured interviews of family physicians in Edmonton, Canada, who experienced conflict with substitute decision-makers of dying patients. Purposeful sampling included maximum variation and theoretical sampling strategies. Interviews were audio-taped, and transcribed verbatim. Transcripts, field notes and memos were coded using the constant-comparative method to identify key concepts until saturation was achieved and a theoretical framework emerged.

**Results:**

Eleven family physicians with a range of 3 to 40 years in clinical practice participated.

The family physicians expressed a desire to achieve a “good death” and described their role in positively influencing the experience of death.

*Finding Common Ground to Achieve a “Good Death” for the Patient* emerged as an important process which includes 1) *Building Mutual Trust and Rapport* through identifying key players and delivering manageable amounts of information*,* 2) *Understanding One Another* through active listening and ultimately, and 3) *Making Informed, Shared Decisions.* Facilitators and barriers to achieving *Common Ground* were identified. Barriers were linked to conflict. The inability to resolve an overt conflict may lead to an impasse at any point. A process for *Resolving an Impasse* is described.

**Conclusions:**

A novel framework for developing *Common Ground* to manage conflicts during end-of-life decision-making discussions may assist in achieving a “good death”. These results could aid in educating physicians, learners, and the public on how to achieve productive collaborative relationships during end-of-life decision-making for dying patients, and ultimately improve their deaths.

## Background

Substitute decision-makers are integral to the care of dying patients and these decision-makers make many healthcare decisions for patients [1]. Conflict of healthcare providers with substitute decision-makers is not uncommon [2,3]. These conflicts can involve families feeling pressured to make decisions; feeling their loved one is a burden to healthcare resources [2]; decisions to withdraw or withhold treatment [2]; management decisions [3]; and concerns over who has the right to make decisions [3,4]. Most conflict situations can be distilled down to the presence of a “Calman’s gap” [5]. “Calman’s gap” is the inverse relationship of the discrepancy between a patient’s actual functional status, and his/her expectation of what it should be [5]. The larger the gap, the poorer the quality of life; the smaller the gap, the better the quality of life [5,6]. Neuenschwander et al. [6] adapted this concept of “Calman’s gap” to describe a discrepancy in the family’s understanding or acceptance of the patient’s condition, and their overall expectations.

A conflict between a physician and the surrogate of a dying patient can contribute to a “bad death” experience for the patient and family. A “bad death” could involve having uncontrolled symptoms or distress, a lack of acceptance of the death, the death not being in agreement with the patient’s or family’s wishes, or the family being burdened [7,8]. Patients and families may fear “bad dying” even more than death itself [9].

Conflicts between physicians and surrogate decision-makers can also negatively impact the family members of the dying patient [10,11]. Surviving caregivers of patients with a poorer quality of life at death experienced a poorer quality of life, and a higher risk of developing major depression [12]. This negative ripple effect of a “bad death” may start with the lack of end-of-life preferences being discussed and/or documented [12].

There has been little published on the strategies to prevent or manage conflict deemed useful by family physicians in their unique position as primary care physicians who have ongoing relationships with the patient and the family. The purpose of this study is to describe the conflict experiences that family physicians have with substitute decision-makers of dying patients and to identify the factors that may facilitate or hinder the end-of-life decision-making process. This will provide insight on how to best manage these complex situations and may ultimately improve the overall care of dying patients.

## Methods

### Design

To gain a better understanding of family physicians’ experiences of conflict with substitute decision-makers and to develop an approach to address these conflicts, we used Grounded Theory methodology [13]. Grounded theory develops an understanding of a problem, and delves into the process to determine how the problem can be resolved [14].

This study received ethical approval from the Health Research Ethics Board Panel B of the University of Alberta, Edmonton, Canada.

### Study setting and sample

The research team consisted of two family physicians in Edmonton, Alberta. The principal investigator has a special interest in Palliative Medicine, and the other investigator has expertise with grounded theory. Both have experience managing conflict with surrogates of dying patients. The recruitment letter was sent by email to 28 potential Edmonton-based English-speaking family physicians to enlist physicians who experienced conflict with surrogates of dying patients in any clinical setting within the past five years. Interested physicians who met the inclusion criteria, were sent an information letter and consent form.

Purposeful sampling sought a variation in the sample for such factors as years in practice, gender, location, and clinical practice type [15]. Sample variation identifies common themes that transcends a focussed sample [15]. Theoretical sampling was also used to provide further insights on the evolving understanding obtained during data analysis [13].

The inclusion criteria included experience of conflict with substitute decision-makers to ensure that the sample was appropriate, since participants would have knowledge of the research topic [16].

We had sought to interview 12 subjects, as the literature shows that samples of 5–20 are adequate in qualitative studies [15]. Category and theoretical saturation [13] was achieved by the eighth participant interviewed, since no new information on key themes was identified in the later interviews.

Our final sample group (Table 1) included 11 family physicians who had a variety of practice experiences. These physicians ranged from 3 to 40 years in clinical practice.

**Table 1:** Demographics of study participants

**Study participant*(RANDOM ORDER)***	**Gender**	**Type of practice**	**Type(s) of location**	**Medical school graduation year**	**Years in practice**
**1**	M	Private clinic, nursing home, home	Rural & urban	1996	11
**2**	F	Academic clinic, hospital, home visits	Urban	2000	8
**3**	M	Private clinic, hospital, community clinic	Urban	2002	6
**4**	F	Academic clinic, hospital, hospice, home visits	Urban & rural	2002	6
**5**	F	Academic clinic, hospital, hospice	Urban	1998	10
**6**	M	Private clinic, hospice, home visits	Urban	1978	32
**7**	F	Private clinic, hospital, hospice, nursing home, home visits	Urban	1988	20
**8**	F	Private clinic, hospital	Urban	1995	13
**9**	M	Academic clinic, hospital, hospice, home visits	Urban & rural	1977	32
**10**	F	Academic clinic, private clinic, hospital, home visits	Urban	2004	3
**11**	M	Academic clinic, private clinic, hospital, hospice, home visits	Urban	1969	40

### Data collection

Individual semi-structured interviews were used in this study because of the sensitive nature of the topics being discussed [17], and to elicit case-oriented narratives and deeper exploration of developing themes [18].

An open-ended interview guide for the semi-structured interviews was developed to ensure key areas were explored, based on the researchers’ previous clinical experiences and the introductory literature review. The draft interview guide was pilot-tested on colleagues who were not participating in the study. The initial question was: “*Could you please tell me in an anonymous manner, about the time(s) when you experienced conflict during an end-of-life decision-making discussion with a substitute decision-maker of a dying patient?*”

The first author conducted each semi-structured interview in person. The interviews took forty-five minutes to seventy-five minutes to complete, and were audio-taped, transcribed verbatim and checked for accuracy.

Thorough field notes were taken after each interview to capture key verbal and nonverbal communications and observations [17]. A journal was kept to assist with documenting the audit trail and included memorandums about possible linkages between data; emerging or contradictory areas that needed further exploration; and the researchers’ evolving perceptions/understandings and potential biases [17].

### Data analysis

The transcripts, field notes and journal memorandums were analyzed manually for emerging themes and key quotes [19]. Analysis was done concurrently with data collection, using an iterative analysis technique, so that future interviews were shaped by the themes identified in prior interviews. Each investigator read and coded each interview transcript separately and then met regularly to review and compare the themes and concepts generated. Differing perspectives were discussed and challenged and in some cases explored in future interviews to gather new information that developed a deeper understanding and achieved consensus that moved beyond the initial individual perspectives. Someone outside the medical field also coded transcript excerpts to confirm the initial coding of themes. An audit trail was created throughout the data collection and analysis stages to help with the constant comparison of data. Memorandums helped to analytically interpret the data, including emerging concepts and relationships as they emerged. Through sorting the memorandums into different groupings, and constantly comparing how each memorandum related to another [13], relationships emerged between the different concepts, giving the categories dimension and position within a theoretical framework [19,20].

The software program, NVivo 8 [21] was used after the manual coding stage to aid in the management of the qualitative data.

### Rigour of study methods

Several methods were used to ensure the rigour, validity, and reliability of this study [22]. Triangulation was achieved through field notes to capture observations not captured in the audiotapes, thereby gathering data from more than one source to ensure comprehensiveness [17,22,23]. Triangulation was also achieved through theoretical sampling [13]. An audit trail was created throughout the data collection. “Member checking”, whereby the findings were verified by some of the participants, was also completed to ensure credibility of the data analysis [17,22,23].

## Results

The family physicians expressed a desire to achieve a “good death” and described their role in positively influencing that experience of death for their patients. They were concerned primarily that any conflict with substitute decision-makers would hinder their ability to help their patients achieve this.

“…although we can’t change the ultimate end point, I think we can change the journey to the end point and I think that’s very powerful and very important in Family Medicine and probably needs to be emphasized.”

**Figure 1:**
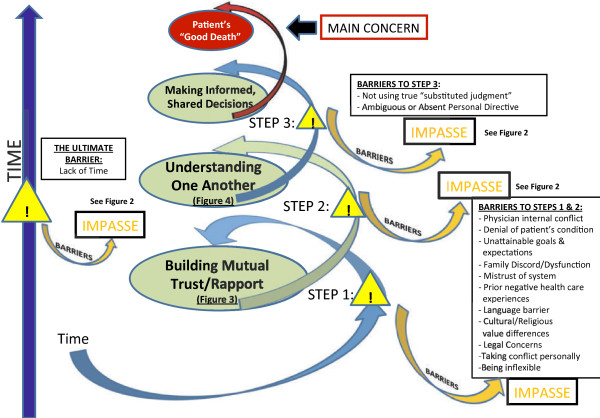
Finding common ground to achieve a “good death” for the patient.

*Finding Common Ground to Achieve a “Good Death” for the Patient*, (Figure 1) emerged as an approach to managing conflict, and to achieving that good death. The three key components in this process that facilitate a patient’s “good death” were identified as: 1) *Building Mutual Trust and Rapport,* 2) *Understanding One Another* and 3) *Making Informed, Shared Decisions* (Figure 1). This iterative process involves going back and forth as necessary. As each layer of the foundation is built, the process of finding *Common Ground* has fewer barriers to overcome to achieve the mutual goal of a “good death”.

**Figure 2:**
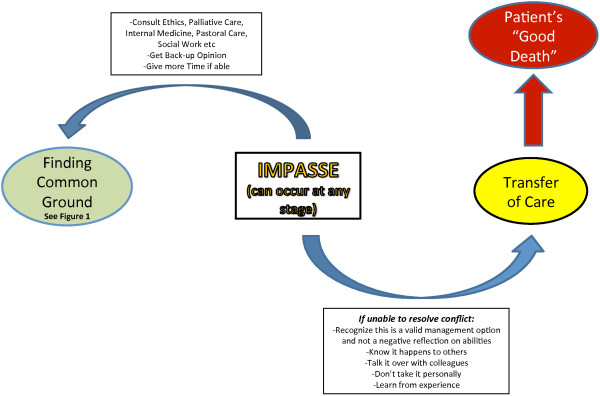
When common ground cannot be achieved – resolving an “impasse”.

Facilitators and barriers to this process were identified. Barriers to *Finding Common Ground* contributed to the conflict in these end-of-life discussions. The inability to resolve an overt conflict may lead to an impasse at any stage of this process. A process for *Resolving an Impasse* is described (Figure 2).

### The process of *finding common ground to achieve a “good death” for the patient*

#### Component 1: Building mutual trust and rapport

**Figure 3:**
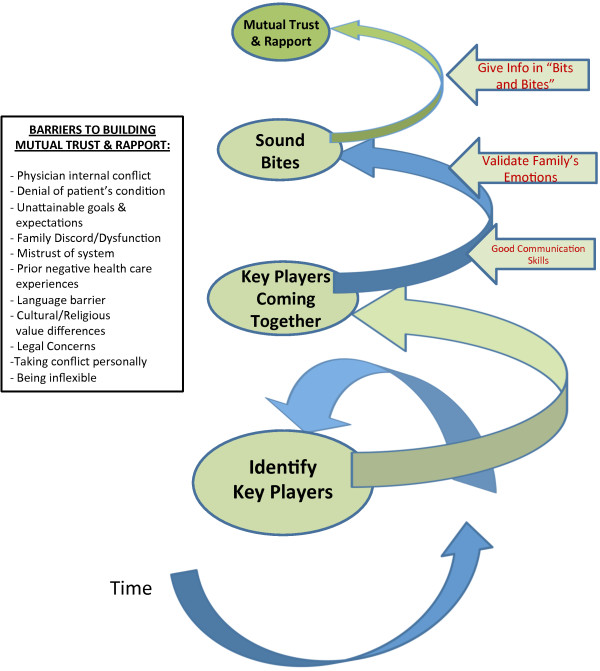
Building mutual trust and rapport.

Through *Building Mutual Trust and Rapport* (Figure 3), roles are clarified and key players come together as bits of information are shared in manageable quantities with multiple contacts over time. The key players include the physician, other members of the multidisciplinary team (such as nurse practitioners, nurses, social workers, chaplains, physiotherapists, respiratory therapists, and dieticians, who can be involved in both the inpatient and outpatient settings) and the key surrogates, as ideally identified by the patient. Compassionate delivery of difficult information to surrogates is essential. Normalizing and checking in on the family’s emotions is especially valuable in building a trusting relationship with surrogates.

“So I think you can enable the patients and families to digest things in smaller chunks so you can basically give them more information over time, and you see them over time, and they have a trust in you…to come to a better understanding of things.”

#### Component 2: Understanding one another

**Figure 4:**
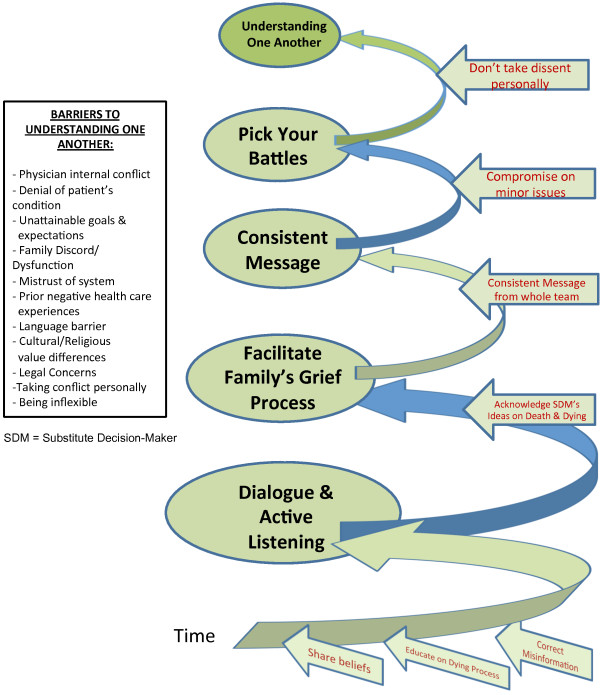
Understanding one another.

Once *Mutual Trust and Rapport* is established, the next major step is *Understanding One Another*. Once people feel understood, they are better able to listen to others, as opposed to focussing on being heard. *Understanding One Another* (Figure 4) entails each participant advocating for the patient while actively listening and educating each other about their respective opinions. Misconceptions are clarified and corrected. The physician also facilitates the surrogates’ grief process and navigates the family through the dying process and the medical system.

“And, tell me a little bit about…what your understanding is of what’s going on here and what are your sorts of thoughts about what’s going to happen now?

… then I learn kind of where we’re at.”

#### Component 3: Making informed, shared decisions

With the establishment of trust and the ability to understand one another, a productive relationship is developed to make informed decisions together to best enable the patient’s “good death”.

After the death of their loved one, the family will be left to live with their decisions. Exploring perceptions on how decisions will affect them after the death may help inform those involved.

“And I think… people need to think about the dying person’s wishes, but ultimately when that person’s gone, you still have to go on and they would want you to go on and be happy, so how do you think this would affect you?”

A physician’s previous discussion about the patient’s wishes when the patient was competent, and clear documentation of this conversation, can help the family understand the patient’s wishes and take some of the pressure off of them.

“This is a very difficult situation that you’re in, having to make a decision for your loved one when they’re not able to tell you what they want, and that’s a lot of pressure on you, but actually we have this to guide us and help us out.”

### Key barriers to the process of *finding common ground to achieve a “good death” for the patient*

Barriers were described that could impede the process of *Finding Common Ground* (Figure 1) leading to conflict and possibly resulting in an impasse (Figure 2).

The physician’s own internal conflict may impede delivering a consistent message to the family. Hence, the physician must first come to terms with understanding and accepting the patient’s prognosis, illness trajectory and best medical management plan.

The family’s denial of the patient’s terminal illness makes it difficult for surrogates to be receptive to information about realistic management options. There may be an unrealistic expectation of what medicine can do.

"“The wife wasn’t really grasping it and probably in some denial…so she was sort of saying, ‘Can we do this? Can we do this? Can we do more?”"

“I think a lot of it has to do with unrealistic expectations for the patients and family though…They expect of medicine what medicine cannot do..”

The lack of a prior relationship between the physician and the dying patient and family means that this relationship is beginning at a very intense and emotional time.

“…because I take on “orphan” palliative patients a lot of the time, you’re meeting people for the first time at precisely the most emotionally stressful time of the patient and usually the family’s life… the potential for me for conflict is greater when I’m coming in as a new physician”

Another major barrier is if there has not been any previous *effective* advance care planning by the patient and family. Family physicians are in the best position to facilitate the discussion about end-of-life care goals and wishes with patients.

“So I really think it is our responsibility, first and foremost, we are the people that know them the best. We are the people that can have this discussion and we’ve got the continuity and the longevity. We know how to bring this up, we know when to bring it up…”

“It really has to be the family physician…in an ideal world, it would always be brought up by the family physician and we would have clear understandings about future wishes of patients.”

### The overarching concept of *time* to the process of *finding common ground*

*Time* is the ultimate facilitator and allows the progression through the three steps of the framework leading to *Common Ground* (Figure 1)*.*

“It takes time. I think understanding the perspective of the substitute decision-maker, or even the patient. And time. And that whole thing of finding common ground, I think is important. And it takes time to find that common ground.”

*Time* is also the ultimate barrier since the overall time for a patient’s clinical decline is ultimately out of everyone’s hands. Physicians, other healthcare team members, and surrogates need to get to *Common Ground* as efficiently as possible.

### Part Two: *Resolving an “Impasse”*

There are times where an “impasse” may occur between the surrogates and the family physician due to unresolvable conflict (Figure 2).

Several strategies emerged as helpful to manage an impasse so as to still achieve a “good death”. One strategy involves seeking a second opinion from members of the multidisciplinary healthcare team, such as nurses, social workers, and chaplains, to try to move back to the process of trying to build some *Common Ground*.

“don’t think that you’re by yourself in these situations…If you ever feel that you’re coming into conflict with someone, always just ask for help and get different perspectives on situations and different ways of dealing with things… don’t ever get angry with it. You know, just stop the conversation if you feel like you’re not getting anywhere, and leave and ask for help.”

There may come a point, however, where the physician may either feel that the conflict is potentially compromising the patient’s care, or *Common Ground* is not achievable. In these instances, transferring the patient’s care to a colleague may be the best course of action for everyone concerned, and improve the outcome towards a “good death”. Changing physicians may bring a new perspective and dynamic. Family physicians who have had to transfer care because of an impasse realized this was a valid treatment option and not a negative reflection of their skills or abilities.

“knowing that one, I was able to transfer care, like I was able to kind of just let go of it at that point, and also try not to internalize it too much, and realize that a lot of the issues were a product of the situation and not something that I had failed on or I had produced or caused, and sort of learn from it rather than use it to kind of flail myself with or sort of feel like I was not doing a good enough job. But that takes a while, right?”

*Experience* was the key to helping family physicians cope with conflict with substitute decision-makers. Experience benefits the physician trying to prevent overt conflict, resolve a conflict situation, or deal with an impasse. These incidents are valuable experiential learning opportunities.

“conflict, dealing with conflicts, I think, makes you more grounded, makes you more experienced to deal with these kind of situations in the future. That’s how I feel…I learn a lot. We all learn a lot from conflicts.”

## Discussion

Family physicians can work to achieve *Common Ground* with substitute decision-makers of dying patients as a means to prevent and/or manage conflict and facilitate the mutual goal of achieving a “good death” experience for everyone involved. Through our exploration of family physicians’ conflict with substitute decision-makers, we developed a cohesive, practical approach that could assist clinicians with finding *Common Ground* with surrogates when providing care to dying patients. “Common Ground” is a key element of the Patient-Centered Clinical Method in Family Medicine as conceptualized by McWhinney [24]. He describes “finding Common Ground” as a “process of clarifying issues, encouraging the patient’s questions, and seeking his or her agreement with the plan” [24]. The specific facilitators and barriers identified for each step in our framework for managing conflict with substitute decision-makers of dying patients was found to fit McWhinney’s more general description of finding common ground within the Patient-Centered Clinical Method [24]. This further supports our framework since entering these various indicators and concepts into our framework lead to the same core variable of *Finding Common Ground*. This supports the Glaserian grounded theory concept of “inter-changeability of indicators” since other indicators can be incorporated or explained by our framework, indicating that the framework is relatively complete [25].

Our framework emphasizes that this process of achieving *Common Ground* relies on time as the ultimate facilitator. Multiple contacts with the surrogates are required to deliver and discuss medical information in manageable quantities. Multiple contacts over time also foster the formation of a trusting relationship between the physician and the surrogates so that each can start to understand each other effectively. There is great benefit to facilitating the family and substitute decision-makers through their grief process to improve the collaborative, shared decision-making process. Not only does the physician need to help surrogates understand the reality of the patient’s medical situation, but also, there needs to be time, acknowledgement, and support given for the family to *grieve* the loss of their hopes for the future with the patient, even prior to the death. As it is the family who will need to be able to live with their decisions beyond the patient’s death, decisions made should not increase their risk of developing future anxiety or depression [10,11]. Thus, embarking on this process to achieve *Common Ground* should be initiated at the first contact of the healthcare team with any surrogate, ideally *before* any conflict has arisen. Each of these detailed elements provide the foundation for the physician, the healthcare team, and substitute decision-makers to work together effectively, and prevent or manage any conflicts so as to make informed decisions which will optimize the achievement of the patient’s “good death”.

Unfortunately, just as time is the ultimate facilitator for this process, it is also the ultimate barrier. At any point of this process, a lack of time prevents conflicts from being effectively resolved, and an impasse could occur. Further contributing factors to an impasse also emerged from our study and some of these concepts support what has already been described in the literature.

Unrealistic expectations and loved ones’ denial of the patient’s condition were both identified as fundamental barriers to achieving Steps 1 and 2 (Figures 1, 3 and 4). Managing these helps to close “Calman’s gap” as defined earlier [5,6]. This may also be an indicator that both the physician and surrogates are *being understood*. Hence, achieving the ideal situation of matching expectations could facilitate finding *Common Ground*. Matching expectations are indicators of the concept of closing “Calman’s gap” [5,6] which, to some degree, represent our concept of achieving Common Ground. This is again evidence of an inter-changeability of indicators, and supports our findings [25].

Even when other identified barriers to Steps 1 and 2 in Figure 1, such as family discord or dysfunction, mistrust of the medical system, language barriers or cultural value differences (Figures 1, 3 and 4), can be reconciled, there may still be difficulties with decision-making due to absent, or ineffective advance care planning with the patient. A lack of preparedness for the role of a substitute decision-maker, and a lack of clear understanding of using true substituted judgement [26,27], (whereby the surrogate is responsible for making the medical decision that the *patient* would have made), further increases the risk for irresolvable conflict to lead to an impasse. These were identified as the key barriers to achieving Step 3 in Figure 1.

The specific approach to resolving an impasse in this study has not been previously described. This approach to managing an impasse gives permission to consider transferring care as a viable management option, if multiple genuine attempts to resolve conflicts have been unsuccessful. When a physician feels that ongoing conflict may actually either affect patient care, cross a physician’s personal moral boundary, or cause a loss of the affective neutrality imperative in a doctor-patient relationship, a “hand-off” [28] of the patient and the surrogates to another physician could be accepted as an appropriate method of termination of the therapeutic relationship [28]. This allows a fresh start to occur for all involved [28], and may ultimately improve the chances of the end goal of a “good death” to still be accomplished. A large European survey showed that physicians often perceive ethical difficulties related to a patient’s impaired decision-making capacity (94.8%), or disagreement with caregivers over making decisions for incompetent patients (81.2%) [29]. Thus, the framework of how to approach an impasse, with the option of transferring care, may help physicians to better cope with these difficult dilemmas and foster effective relationships with surrogates of dying patients, thereby increasing job satisfaction and decreasing job stress [30].

Our results underscore what has been recommended for the last decade about educating the general public about death, dying, and planning for this eventuality [31]. Richard Smith [32], remarked in a *British Medical Journal* editorial that health services need to change its view of death and dying so as improve the chances for achieving good deaths. He argues: “If death is seen as a failure rather than as an important part of life then individuals are diverted from preparing for it and medicine does not give the attention it should to helping people die a good death” [32]. If society as a whole was better informed about the dying process, conceivably, this would help facilitate more open discussion about end-of-life care and wishes [33]. The framework described in this study may help in educating the public on how to have these discussions effectively with healthcare providers and surrogates, and to not delay these conversations. Perhaps then, terms such as “code status” or “Personal Directives” will not be seen as “icons of death”, but rather an opportunity to delineate one’s definition of a “good death”.

Family physicians are in an ideal position to guide patients through the advance care planning process and to encourage their potential surrogates to actively participate. In the 2004 Ipsos-Reid survey [34] on Canadian Hospice Palliative Care, 44% of Canadians had discussed their EOL wishes with family, and only 9% had discussed these with their physicians. Family physicians can take advantage of their established relationship to determine how best to broach these sensitive end-of-life issues with each patient, and positively influence death experiences for patients and their families. Since primary care physicians or family physicians are best trained to treat the whole person, and to coordinate care within the healthcare system, they are “the best prepared of all physicians to hear and implement patients’ wishes regarding care” [35]. Even when a patient must be transferred to a care facility where the family physician cannot continue to be the primary attending physician, the early work that the family physician may have done with the patient and substitute decision-makers in delineating his/her end-of-life wishes can still be of benefit. “Informational continuity” [36] can result through the direct transfer of this information, including the patient’s values, beliefs and any specific end-of-life preferences, from the family physician to the new attending physician. With this transfer of information, the key foundational work that the family physician will have furthered can then be built upon. The next steps in any conversation regarding a patient’s wishes may then be less overwhelming to the surrogates because the concepts will not be wholly foreign. Thus, *Common Ground* may be attained more efficiently for the next provider. This concept may be generalized to other primary care providers, such as general internists, and primary care nurse practitioners.

A major barrier to pre-emptive advance care planning is the amount of time required to have these conversations with patients and their surrogates. Family physicians need to be financially supported to take the time necessary for these complicated discussions. This seems even more urgent given Canada’s aging population. An Ontario study found that detailed advance care planning can save health care costs in nursing home facilities [37]. This argues for the economic benefits of financially supporting Canadian family physicians to implement detailed advance care planning for all of their patients as part of their comprehensive practice.

### Limitations of study

This study involved family physicians who work only in Northern Alberta, Canada. While the participants work in a variety of clinical settings, the findings of this study may not be applicable in other clinical settings, or different healthcare systems.

Initially, the research team had preconceptions and possible selection bias which may have limited exploring the experiences of those who did not perceive having had conflict. Perhaps those who did not perceive conflict with surrogates have refined skills in preventing or handling conflict.

Ethical constraints inhibited interviews with substitute decision-makers and dying patients to provide further insight.

### Future directions

Future work may include further theoretical sampling with family physicians who self-report not experiencing conflict to gain more insight from their perspectives. Theoretical sampling of intensive care specialists, nephrologists, oncologists and others who work closely with dying patients, to explore the ways in which they manage conflict with surrogates of dying patients may be beneficial. Studies with other primary care providers would further the insights gathered from this study.

Initiatives are necessary to encourage and facilitate family physicians to foster their unique relationships with their patients, and to commence these important end-of-life conversations so as to help their patients achieve “good deaths”. Effective methods to improve “informational continuity” [36] of advance care planning within a healthcare system are required as well.

## Conclusion

Conflict between physicians and substitute decision-makers of dying patients can occur for a multitude of reasons, and can potentially contribute to a patient’s “bad death” with ramifications for everyone involved. A novel framework for developing *Common Ground* is described to help resolve these conflicts, and may assist in achieving a “good death”. These results may aid in educating physicians, learners, and the public on how to have productive collaborative relationships during end-of-life decision-making for dying patients, and ultimately improve their deaths.

## Competing interests

The authors declare that they have no competing interests.

## Authors’ contributions

AT conceived of the study, participated in its design and coordination, conducted the interviews, analyzed and interpreted the data, and drafted and revised the manuscript. DM participated in the study design and coordination, analyzed and interpreted the data, helped to develop an outline, and helped draft and revise the manuscript. All authors have read and approved the final manuscript.

## Authors’ informations

AT is an Assistant Professor and Family Physician and Palliative hospice physician in the Department of Family Medicine at the University of Alberta and has a Masters of Science degree in Palliative Medicine from Cardiff University. She is the Undergraduate Education Program Director in the Department of Family Medicine.

DM is an Associate Professor and Family Physician in the Department of Family Medicine at the University of Alberta. She is the Research Director in the Department of Family Medicine and the Clinical Director of the Alberta Family Practice Research Network.

## Pre-publication history

The pre-publication history for this paper can be accessed here:

http://www.biomedcentral.com/1471-2296/14/14/prepub
